# Caffeine and Caffeine Metabolites in Relation to Insulin Resistance and Beta Cell Function in U.S. Adults

**DOI:** 10.3390/nu12061783

**Published:** 2020-06-15

**Authors:** Sohyae Lee, Jin-young Min, Kyoung-bok Min

**Affiliations:** 1 Department of Preventive Medicine, College of Medicine, Seoul National University, Seoul 03080, Korea; leesohyae@snu.ac.kr; 2 Institute of Health and Environment, Seoul National University, Seoul 08826, Korea; yaemin00@snu.ac.kr

**Keywords:** caffeine, xanthines, insulin resistance, beta cell function, cross-sectional study

## Abstract

The relationship between caffeine and insulin resistance (IR) has been assessed only in terms of caffeine intake, and the association between caffeine and beta cell function (BCF) remains unclear. This study examines the association between urinary caffeine and its metabolites, IR, and BCF in nondiabetic, noninstitutionalized US adults in order to account for the inter-individual differences in caffeine metabolism. Data on urinary caffeine and its metabolites, IR and BCF from adults aged 20 years and older who participated in the 2009–2010 and 2011–2012 National Health and Nutrition Examination Surveys were analyzed (*n* for caffeine = 994). IR and BCF were assessed using homeostatic model assessment (HOMA) and urinary caffeine and its metabolites were measured using high-performance liquid chromatography-electrospray ionization-tandem quadrupole mass spectrometry. After adjusting for all covariates, increases in urinary 1,3-DMU, 1,7-DMU, 1,3,7-TMU, theophylline, paraxanthine, caffeine, and AAMU were significantly associated with increased HOMA-IR and HOMA-β (HOMA of insulin resistance and beta cell function). Compared with individuals in the lowest quartile of urinary 1,3-DMU, 1,7-DMU, 1,3,7-TMU, theophylline, paraxanthine, caffeine, and AAMU, the regression coefficients for HOMA-IR and HOMA-β were significantly higher among those in the highest quartile. After stratification by prediabetes status, HOMA-IR and HOMA-β showed significant positive associations with urinary caffeine and its metabolites among subjects with normal fasting plasma glucose levels. Our cross-sectional study showed that caffeine and its metabolites were positively related to IR and BCF.

## 1. Introduction

Diabetes mellitus (DM) is a worldwide public health problem considered one of the four priority noncommunicable diseases targeted by world leaders [1]. Globally, the prevalence of DM has doubled from 1980 from 4.7% to 8.5% and, in 2014, approximately 422 million adults were living with DM [1]. In the United States, 34.2 million adults (13.0%) had DM and 1.5 million new cases were diagnosed in 2018 [2]. Factors such as age, body weight, sex, genetic predisposition, and lifestyle factors such as physical activity and diet are associated with insulin resistance (IR) [3]. In addition to carbohydrates, fibers, and fatty acid composition, an association between caffeine and IR has been reported [3,4,5,6,7].

The association between caffeine consumption and IR has been reported in a diverse population [7,8,9]. Previous studies investigating the relationship between caffeine and IR have reported that acute caffeine intake is associated with an increase in IR [5,6,8,9,10], while chronic coffee consumption is associated with insulin sensitivity and a protective effect against type 2 DM risk [7,11,12,13,14]. Conversely, the relationship between coffee and beta cell function (BCF) has been studied to a lesser extent and shows conflicting results; studies in Japanese adults and men from the Uppsala Longitudinal Study of Adult Men reported no association, while other studies from the Insulin Resistance Atherosclerosis Study (IRAS) and Shanghai High-Risk Diabetic Screen (SHiDS) study reported a positive association [5,15,16,17]. Furthermore, no studies so far have investigated the relationship between caffeine and BCF.

Previous studies on the relationship between caffeine and IR have assessed caffeine exposure only in terms of caffeine intake [6,8,18]. Although food frequency questionnaires or 24-h dietary recalls are the source of most estimates of caffeine intake, they are usually limited to beverages and do not capture the full spectrum of caffeine-containing products [19,20]. Additionally, the interindividual differences in caffeine metabolism, which may result in different amounts of circulating caffeine and caffeine metabolites, have not been considered [21].

To account for the inter-individual differences in caffeine metabolism, and in order to study the associations between caffeine and its metabolites, IR and BCF, we sought to determine the association between IR, BCF, and urinary caffeine and its metabolites in a representative sample of nondiabetic, noninstitutionalized US adults using homeostatic model assessment (HOMA) modelling.

## 2. Materials and Methods

### 2.1. Study Population

Data on urinary caffeine and caffeine metabolites, insulin, and fasting glucose were obtained from the 2009–2010 and 2011–2012 National Health and Nutrition Examination Surveys (NHANES), a representative survey of the civilian population in the US [22]. A total of 20,293 people were included from both cycles. Urinary caffeine was measured in 5,112 people. The analysis was restricted to individuals with urinary caffeine and caffeine metabolite levels at or above the lower limits of detection due to the fact that the regression equations, accounting for the possible differences resulting from an instrument change between the two cycles, did not include values below the lower limits of detection [23,24]. As a result, urinary caffeine data was available for a total of 4782 individuals.

The analysis was restricted to adults aged 20 years and older (*n* = 3426) without a history of diabetes, diabetic drug use, or insulin use (*n* = 3009). Among these subjects 1361 were tested for fasting insulin and fasting glucose; however, because the interpretation of HOMA results generated when fasting insulin is less than or equal to 5 uU/mL and fasting glucose is less than 4.5 mmol/L are not valid, the study sample was limited to individuals with fasting insulin levels greater than 5 uU/mL and fasting glucose levels greater than or equal to 4.5 mmol/L (*n* = 1192) [25]. Among these subjects, 994 individuals had data for all covariate variables.

### 2.2. HOMA Modelling

IR was evaluated by HOMA-IR (fasting serum insulin (μIU/mL) ∗ fasting plasma glucose (mmol/L)/22.5) and BCF was evaluated by HOMA-β (20 ∗ fasting serum insulin (μIU/mL)/(fasting plasma glucose (mmol/L) − 3.5)) [26]. Fasting glucose and insulin blood tests were performed on participants after a nine-hour fast. Specimens were stored at −70 °C until analyzed at Fairview Medical Center Laboratory at the University of Minnesota, Minneapolis Minnesota [27,28]. To account for a change in methods for insulin measurements during the NHANES 2009–2010 cycle from the Mercodia sandwich ELISA assay to a Roche chemiluminescent immunoassay performed on the Elecsys 2010 analyzer in late 2009, a fractional polynomial regression was used to trend insulin from the NHANES 2011–2012 cycle to match the NHANES 2009–2010 cycle [27]. Glucose was measured using an enzymatic method measuring the increase in absorbance at 340 nm resulting from the reduction of NADP + to NADPH during the conversion of glucose-6-phosphate to gluconate-6-P by glucose-6-phosphate dehydrogenase [27].

### 2.3. Measurements of Urinary Caffeine and Caffeine Metabolite Levels

Urine samples for the study participants were collected after an overnight fast for 9 h during the morning session [28,29]. Urinary caffeine and caffeine metabolites were measured using high-performance liquid chromatography-electrospray ionization-tandem quadrupole mass spectrometry (HPLC-ESI-MS/MS) with stable isotope labeled internal standards for the NHANES 2009–2010 and NHANES 2011–2012 cycles, but with different instruments for each cycle. To account for the instrument change, regression equations from the bridging study were used when combining data from the two cycles for analysis [24]. Caffeine and 14 of its metabolites were measured: 1-methyluric acid (1-MU), 3-methyluric acid (3-MU), 7-methyluric acid (7-MU), 1,3-dimethyluric acid (1,3-DMU), 1,7-dimethyluric acid (1,7-DMU), 3,7-dimethyluric acid (3,7-DMU), 1,3,7-trimethyluric acid (1,3,7-TMU), 1-methylxanthine (1-MX), 3-methylxanthine (3-MX), 7-methylxanthine (7-MX), 1,3-dimethylxanthine (theophylline, 1,3-DMX), 1,7-dimethylxanthine (paraxanthine, 1,7-DMX), 3,7-dimethylxanthine (theobromine, 3,7-DMX), 1,3,7-trimethylxanthine (caffeine, 1,3,7-TMX), and 5-acetylamino-6-amino-3-methyluracil (AAMU). Among these compounds, we selected metabolites that showed an association with caffeine intake [30]. As a result, caffeine and eight of its metabolites were included in the present analysis: 1-MU, 1,3-DMU, 1,7-DMU, 1,3,7-TMU, 1-MX, 1,3-DMX (theophylline), 1,7-DMX (paraxanthine), 1,3,7-TMX (caffeine), and AAMU.

### 2.4. Other Variables of Interest

Covariates were obtained from the NHANES 2009–2012 and included sex (male or female), age (20–29, 30–39, 40–49, 50–59, 60–69, or 70–79 years), ethnicity (non-Hispanic white, non-Hispanic black, Hispanic, or other), annual family income (less than $20,000 or $20,000 or more), education (less than high school, high school graduate, or more than high school), marital status (married, never married, widowed, divorced, separated, or living with partner), smoking status (never, former smokers, or current smoker), alcohol consumption (yes or no), physical activity (yes or no), body mass index (BMI) (underweight (<18.5 kg/m^2^), normal weight (18.5–24.9 kg/m^2^), overweight (25.0–29.9 kg/m^2^), or obese (>30 kg/m^2^)), urine creatinine (mg/dL), and total caffeine intake (mg/day) from food and beverages per day measured using a 24-h dietary recall interview.

### 2.5. Statistical Analysis

Concentrations of urinary caffeine and caffeine metabolites were analyzed as both continuous and categorical variables categorized into quartiles. Distributions of urinary caffeine and caffeine metabolites were right-skewed and log transformed. Pearson’s correlation coefficients among HOMA-IR, HOMA-β, and urinary caffeine and caffeine metabolites were calculated. Linear regression analysis was performed to determine associations between urinary caffeine and caffeine metabolites, HOMA-IR and HOMA-β. Linear regression analyses provided beta coefficients and standard errors (SE) for HOMA-IR and HOMA-β among individuals with higher levels of urinary caffeine and caffeine metabolites (quartiles 2 through 4) compared to those with the lowest level of urinary compounds (quartile 1) as the reference group. The adjusted linear regression model was adjusted for all potential confounders including age, sex, race, income, education, marital status, smoking history, alcohol consumption, physical activity, BMI, urinary creatinine clearance, and caffeine intake. Linear regression analyses were also performed after stratification for prediabetes status.

According to the NHANES analytic guidelines, weighted estimates of the population parameters were applied to account for the complex sampling design. Analyses were performed with the PROC SURVEY procedures of SAS 9.4 statistical analysis package (SAS Institute, Cary, NC, USA). The statistical significance was set at α = 0.05.

### 2.6. Ethical Approval and Consent to Participate

The present study was exempt from formal ethics review as a secondary analysis of existing NHANES public data under the US Health & Human Services (HHS) regulations at 45 CFR 46.101 (b) [31]. The NHANES surveys were approved by the US National Center for Healthcare Statistics (NCHS) Research Ethics Review Board (ERB), and the NCHS IRB/ERB protocol number for NHANES 2009–2010 was a continuation of #2005-06 and NHANES 2011–2012 was #2011-17 [32]. Participants gave written informed consent before the home interview and health exams [33].

## 3. Results

The characteristics of the study participants with data for urinary caffeine are presented in Table 1. The mean age of participants was 48.0 (± 17.5) years (range: 20–80 years), and women represented 50.5% of the overall sample. Most of the participants were non-Hispanic white (47.7%), not impoverished (76.2%), and had an education beyond high school (53.5%). A majority of the participants were overweight (35.9%) or obese (38.7%). Mean caffeine intake was 163.0 (± 197.7) mg/day, with caffeine intake from coffee (mean ± SD: 105.3 ± 173.3 mg/day) being the highest followed by tea (25.7 ± 82.5 mg/day), soda (25.4 ± 49.0 mg/day), and energy drinks (3.3 ± 24.5 mg/day).

The sample sizes, distribution, and geometric means of the urinary concentrations of caffeine and caffeine metabolites are shown in Table 2. Concentrations for 1-MU were the highest with a geometric mean (standard error) value of 62.06 (±3.22) umol/L followed by AAMU, 1-MX, 1,7-DMU, 1,7-DMX, 1,3-DMU, 1,3,7-TMX, 1,3-DMX, and 1,3-TMU in decreasing order.

Table 3 shows the Pearson’s correlation coefficients between all variables. Statistically significant (*p* < 0.05) positive correlations with correlation coefficients were observed between all variables.

Table 4 shows the beta coefficients for the associations between urinary caffeine and caffeine metabolite levels and HOMA-IR and HOMA-β. HOMA-IR and HOMA-β scores showed a significant positive association with all binary logarithm transformed urinary caffeine and caffeine metabolite levels in the unadjusted models and with 1,3-DMU, 1,7-DMU, 1,3,7-TMU, 1,3-DMX, 1,7-DMX, 1,3,7-TMX, and AAMU after adjusting for all covariates. In the adjusted model, a two-fold increase in 1,3-DMU, 1,7-DMU, 1,3,7-TMU, 1,3-DMX, 1,7-DMX, 1,3,7-TMX, and AAMU was associated with a 0.16 (SE = 0.07, *p*-value 0.027), 0.18 (SE = 0.06, *p*-value 0.005), 0.25 (SE = 0.07, *p*-value 0.001), 0.21 (SE = 0.07, *p*-value 0.007), 0.21 (SE = 0.06, *p*-value 0.003), 0.24 (SE = 0.06, *p*-value < 0.001), and 0.15 (SE = 0.07, *p*-value 0.037) increase in HOMA-IR, respectively. For the adjusted model for HOMA-β, a two-fold increase in 1,3-DMU, 1,7-DMU, 1,3,7-TMU, 1,3-DMX, 1,7-DMX, 1,3,7-TMX, and AAMU was associated with a 4.23 (SE = 1.49, *p*-value 0.008), 4.08 (SE = 1.35, *p*-value 0.005), 5.62 (SE = 1.59, *p*-value 0.001), 5.40 (SE = 1.50, *p*-value 0.001), 5.73 (SE = 1.53, *p*-value 0.001), 5.46 (SE = 1.57, *p*-value 0.002), and 3.97 (SE = 1.62, *p*-value 0.020) increase in HOMA- β, respectively. Associations with HOMA-β yielded higher beta coefficients compared to HOMA-IR.

Beta coefficients for the associations between HOMA-IR, HOMA-β, and quartiles of 1,3-DMU, 1,7-DMU, 1,3,7-TMU, 1-MX, 1,3-DMX, 1,7-DMX, 1,3,7-TMX, and AAMU showed positive associations with varying significance both before and after adjustment (excluding a negative association for the adjusted model of quartile 2 of AAMU and HOMA-IR). Most beta coefficients showed an increasing dose-response relationship from quartile 2 to quartile 4 compared to quartile 1 as the reference; however, in the adjusted HOMA-IR model for 1,7-DMU and 1-MX, unadjusted HOMA-β model for 1,3,7-TMU and 1,7-DMX, and adjusted HOMA–β model for 1,7-DMU, 1,3,7-TMU, 1-MX, and 1,7-DMX the beta coefficients showed a decrease from quartile 2 to quartile 3, followed by an increase from quartile 3 to 4, compared to quartile 1 as the reference. Individuals in the highest quartile of all urinary compounds (Q4), excluding 1-MX in the adjusted model and 1-MU, showed significant positive beta coefficients, compared to individuals in the lowest quartile (Q1) as a reference, in both the adjusted and unadjusted models of HOMA-IR and HOMA-β. Quartiles 2 or 3 of 1,3-DMU, 1,3,7-TMU, 1,3-DMX, 1,7-DMX, and 1,3,7-TMX additionally showed significant beta coefficients: for 1,3-DMU quartile 3 in both unadjusted models; for 1,3,7-TMU quartile 3 in the both HOMA-IR models and the unadjusted HOMA-β model, and quartile 2 in both HOMA-β models; for 1,3-DMX quartile 2 in the adjusted HOMA-β model and quartile 3 in all four models; for 1,7-DMX quartile 3 in both HOMA-IR models and quartile 2 and 3 in the adjusted HOMA-β model; and for 1,3,7 TMX quartile 3 in all four models in addition to quartile 2 in both adjusted models showed a significant association compared to quartile 1 as the reference.

Table 5 shows the beta coefficients for HOMR-IR and HOMA-β by two-fold increases in urinary caffeine and caffeine metabolites stratified by prediabetes status. For individuals with normal fasting plasma glucose levels, a two-fold increase in all urinary metabolites, excluding 1-MU, showed significant positive associations with HOMA-IR in the adjusted model and with HOMA-β in both models. In contrast, for individuals with prediabetes, significant associations were only observed for the association between 1,7-DMU, 1,3,7-TMU, theophylline, paraxanthine, and caffeine, and HOMA-IR in the unadjusted model; 1,3,7-TMU and caffeine, and HOMA-IR in the adjusted model; and 1,3,7-TMU, paraxanthine, and AAMU, and HOMA-β in the unadjusted model. Overall, the beta coefficients for HOMA-IR and urinary compounds were generally higher among those with prediabetes, while the beta coefficients for HOMA-β and urinary caffeine and its metabolites were generally higher among those with normal fasting plasma glucose levels.

## 4. Discussion

In this study, we found that urinary levels of caffeine and its metabolites were positively associated with IR and BCF in nondiabetic adults. Two-fold increases in urinary caffeine and caffeine metabolite levels, excluding 1-MU in the adjusted models, showed significant positive associations with HOMA-IR and HOMA-β scores, and the highest levels of urinary 1,3-DMU, 1,7-DMU, 1,3,7-TMU, 1,3-DMX, 1,7-DMX, 1,3,7-TMX, and AAMU (quartile 4) showed significant positive regression coefficients, greater than quartile 2 and 3, compared to quartile 1 as the reference.

Our results regarding IR are consistent with results from a previous cross-sectional study which reported that high serum caffeine and paraxanthine concentrations were associated with HOMA-IR in fasting subjects enrolled in the Pregnancy Exposures and Preeclampsia Prevention Study (PEPP) [34]. With the exception of this cross-sectional study, this is the first study examining the relationship caffeine and its metabolites and IR. However, previous studies have reported a positive association between acute caffeine intake and IR [6,8,9,10,35,36,37]. A recent systematic review by Emami et al., (2019) revealed that acute caffeine supplementation increases glucose concentration (weighted mean difference (WMD) = 2.48, 95% CI: 0.75 to 4.20; *p* = 0.005) and decreases insulin sensitivity indices (WMD = 0.03, 95% CI: −0.84 to 0.89) in both healthy subjects and those with type 2 DM [8]. In contrast, chronic intake has been associated with insulin sensitivity and a reduced type 2 DM risk [5,7,10,11,12,13,14]. A cross-sectional study from the Uppsala Longitudinal Study of Adult Men (ULSAM) revealed a positive association between coffee and tea consumption and insulin sensitivity, showing that long-term consumption may show a different metabolic effect compared to short-term caffeine consumption [5]. A study in rats also showed that chronic caffeine intake reversed age-induced IR [38]. Additionally, studies have shown that both caffeinated and decaffeinated coffee are associated with a decreased risk of type 2 DM, revealing that constituents of coffee other than caffeine such as chlorogenic acid, polyphenols, and lignin may be responsible for coffee’s protective role in type 2 DM [39,40,41].

Some of the most common mechanisms regarding the effects of caffeine on IR include increases in adenosine receptor antagonism and catecholamine release [42]. An animal study testing the effects of caffeine on insulin sensitivity of skeletal muscles revealed that acute caffeine administration decreased insulin sensitivity in a dose-dependent manner mediated by A1 and A2B adenosine receptors [43]. Caffeine is also known to stimulate the release of catecholamines, especially epinephrine, which impairs both hepatic and peripheral insulin sensitivity [6,44]. Metabolites of caffeine including paraxanthine, theobromine, and theophylline are biologically active and may play a role in causing IR [21]. An experimental study in humans showed that caffeine and paraxanthine have similar sympathomimetic actions producing an increase in diastolic blood pressure, plasma epinephrine levels and free fatty acids, and paraxanthine is as potent as caffeine in blocking adenosine receptors [21,45]. In line with these results, 1,3,7-TMX (caffeine), 1,3-DMX (theophylline) and 1,7-DMX (paraxanthine) showed positive associations with HOMA-IR in our study. However, no previous studies have considered 1,3-DMU, 1,7-DMU, 1,3,7-TMU, and AAMU in relation to IR. As a result, it is difficult to compare our results with those of previous studies.

The mean fasting plasma glucose level for the study participants was in the range of prediabetes (5.6–6.9 mmol/L). In order to find out whether fasting plasma glucose levels influenced the results, we conducted linear regression analyses after stratifying for fasting plasma glucose. Significant positive associations between HOMA-IR, HOMA-β, and urinary caffeine and its metabolites were consistently observed among individuals with normal fasting glucose levels. In contrast, the regression coefficients were generally less significant among individuals with prediabetes, and further studies should be conducted in order to clarify this relationship.

We also observed a positive association between caffeine and its metabolites and BCF; however, evidence on the association between coffee intake and BCF is limited and inconsistent. A cross-sectional study in Japanese men showed that coffee consumption was significantly, inversely associated with HOMA-IR but showed no association with HOMA-β [17]. A cross-sectional study from the Uppsala Longitudinal Study of Adult Men (ULSAM) showed that coffee consumption was associated with an increase in insulin sensitivity but not with insulin secretion measured as the early insulin response, attributing the proposed antidiabetogenic effect of coffee as the effect of increased insulin sensitivity rather than improved BCF [5]. A study from the Insulin Resistance Atherosclerosis Study (IRAS) reported that decaffeinated coffee, but not caffeinated coffee, was favorably related to measures of BCF, measured by the intact and split proinsulin to C-peptide ratios, and a cross-sectional study in Sweden showed that there was an association between high coffee consumption and beta cell function in those with diabetes and impaired glucose tolerance, but not in subjects with normal glucose tolerance [4,16]. In contrast, a recent study in a large high-risk diabetic Chinese population showed that coffee intake is positively related to BCF measured using HOMA-β [odds ratio (OR) = 2.270, 95% CI: 1.456–3.538] [15]. Evidence on the relationship between coffee intake and BCF is inconsistent and remains limited, and furthermore, the association between caffeine and BCF remains unclear. Studies in mouse islet grafts showed that glucose-induced insulin secretion was potentiated by caffeine and in a randomized controlled trial of nine healthy fasted volunteers, insulin concentrations were higher after caffeinated coffee consumption than after decaffeinated coffee consumption (*p* < 0.05) [46,47]. Accordingly, caffeine or its metabolites, but not coffee, may be what is actually associated with increased BCF. Our results showed that caffeine may show an antidiabetogenic effect via increased BCF, in contrast to a previous study by Ärnlöv et al., (2004) which proposed that the antidiabetogenic effect of coffee may involve insulin sensitivity rather than improved BCF [5]. Further studies are recommended to clarify the association between coffee, caffeine, and BCF.

We have accounted for the inter-individual differences in caffeine metabolism by investigating the association of caffeine and caffeine metabolites and IR and BCF in a representative sample of nondiabetic, noninstitutionalized US adults. Nevertheless, our study has several limitations. Firstly, due to its cross-sectional nature we cannot confirm any casual or temporal relationships. In addition, due to the nature of the NHANES data, we were unable to account for the differences in acute and chronic consumers. Further studies should address the differences between acute and chronic consumption in relation to HOMA-IR, HOMA-β, and urinary caffeine and its metabolites. Secondly, BCF and IR was assessed using HOMA modelling; however, HOMA modelling has been shown to correlate well with established methods such as hyperglycaemic and euglycaemic clamps [26,48]. Thirdly, the relationship between HOMA-IR, HOMA-β, and caffeine and its metabolites may not necessarily be linear. As a result, the associations found in this study may not be generalized to populations with different ranges of urinary caffeine and caffeine metabolites. Fourthly, although basic sociodemographic confounding variables were considered, not all possible confounding variables may have been controlled for. Some studies have also included waist circumference, total energy intake and a family history of diabetes as confounding variables [5,15,16]. Therefore, we cannot exclude the possibility of residual confounding. Fifthly, serum levels of caffeine and its metabolites have not been measured. Despite the fact that urinary caffeine and its metabolites are associated with caffeine intake, urinary levels may not precisely reflect serum levels due to the fact that caffeine is reabsorbed from the renal tubule to reach equilibrium with unbound plasma caffeine [30,49]. Further studies evaluating the relationship between plasma caffeine and caffeine metabolite levels are recommended.

## 5. Conclusions

In conclusion, our cross-sectional study showed that caffeine and its metabolites were positively related to IR and BCF using HOMA modelling, suggesting that caffeine may play a protective role in T2DM via increased BCF. Further studies should be conducted (1) to investigate the effects of individual metabolites on BCF and IR, and (2) to clarify the relationship and mechanisms behind coffee, caffeine, and beta cell function.

## Figures and Tables

**Table 1:** Characteristics of study participants.

Characteristics	Mean ± SD or N (%)
No of participants	994
Sex	
Male	492 (49.5%)
Female	502 (50.5%)
Age at interview (year)	48.0 ± 17.5
Age categories	
20–29	186 (18.7%)
30–39	171 (17.2%)
40–49	182 (18.3%)
50–59	167 (16.8%)
60–69	141 (14.2%)
70–79	147 (14.8%)
Ethnicity	
Non-Hispanic white	474 (47.7%)
Non-Hispanic black	182 (18.3%)
Hispanic	257 (25.9%)
Others	81 (8.1%)
Annual Family Income	
Less than $20,000	237 (23.8%)
$20,000 and over	757 (76.2%)
Education	
Less than high school	242 (24.4%)
High school graduate	220 (22.1%)
More than high school	532 (53.5%)
Marital status	
Married	514 (51.7%)
Never married	194 (19.5%)
Widowed/divorced/separated	286 (28.8%)
Smoking history	
Never smoked	558 (56.1%)
Ex-Smoker	240 (24.1%)
Current Smoker	196 (19.7%)
Alcohol consumption ^1^	
Yes	742 (74.6%)
No	252 (25.4%)
Physical Activity ^2^	
Yes	419 (42.2%)
No	575 (57.8%)
BMI (kg/m^2^)	
Underweight (<18.5)	7 (0.7%)
Normal weight (18.5–24.9)	245 (24.6%)
Overweight (25.0–29.9)	357 (35.9%)
Obesity (>30)	385 (38.7%)
Urine creatinine (mg/dL)	129.6 ± 79.3
Fasting glucose, mmol/L	5.7 ± 1.0
Fasting insulin, uU/mL	15.5 ± 11.7
HOMA-IR	4.1 ± 3.7
HOMA-B	148.8 ± 101.7
Total caffeine intake (mg/day)	163.0 ± 197.7
Caffeine intake from coffee	105.3 ± 173.3
Caffeine intake from tea	25.7 ± 82.5
Caffeine intake from soda	25.4 ± 49.0
Caffeine intake from energy drinks	3.3 ± 24.5

^1^ Response to the question: “In any one year, have you had at least 12 drinks of any type of alcoholic beverage?” ^2^ Response to the question: “In a typical week do you do any moderate-intensity sports, fitness, or recreational activities that cause a small increase in breathing or heart rate such as brisk walking, bicycling, swimming, or volleyball for at least 10 min continuously?”

**Table 2:** Means and Distribution percentiles of urine concentrations of different metabolites (units umol/L).

Urinary Metabolite	*n*	Geometric Mean ± SE	*p*10	*p*25	*p*50	*p*75	*p*90
1-methyluric acid	1036	62.06 ± 3.22	11.30	25.30	60.80	135.00	276.00
1,3-dimethyluric acid	1016	7.08 ± 0.44	0.82	2.97	7.73	16.97	33.40
1,7-dimethyluric acid	1020	23.87 ± 1.52	2.67	9.96	27.44	65.95	127.88
1,3,7-trimethyluric acid	980	1.19 ± 0.08	0.14	0.45	1.41	3.40	6.80
1-methylxanthine	1036	26.86 ± 1.40	3.01	11.10	28.60	66.17	128.62
1,3-dimethylxanthine (theophylline)	1002	1.69 ± 0.09	0.28	0.80	1.90	3.80	6.56
1,7-dimethylxanthine (paraxanthine)	1019	12.88 ± 0.74	1.72	6.36	15.90	32.20	55.90
1,3,7-trimethylxanthine (caffeine)	994	2.71 ± 0.17	0.36	1.10	3.09	7.02	12.98
5-acetylamino-6-amino-3-methyluracil	1027	60.38 ± 3.67	7.94	24.76	63.20	155.00	296.87

**Table 3:** Pearson correlation coefficients among HOMA-IR, HOMA-B (homeostatic model assessment of insulin resistance and beta cell function, respectively), and log-transformed urinary caffeine and caffeine metabolites in the study population.

		HOMA-IR	HOMA-B	1-MU	1,3-DMU	1,7-DMU	1,3,7-TMU	1-MX	1,3-DMX	1,7-DMX	1,3,7-TMX	AAMU
**HOMA-IR**	*r*	1.00										
	*p*											
**HOMA-B**	*r*	0.66	1.00									
	*p*	<0.0001										
**1-MU**	*r*	0.08	0.06	1.00								
	*p*	0.0138	0.0397									
**1,3-DMU**	*r*	0.10	0.08	0.91	1.00							
	*p*	0.0015	0.0153	<0.0001								
**1,7-DMU**	*r*	0.12	0.09	0.87	0.95	1.00						
	*p*	0.0001	0.0043	<0.0001	<0.0001							
**1,3,7-TMU**	*r*	0.15	0.11	0.79	0.89	0.94	1.00					
	*p*	<0.0001	0.0004	<0.0001	<0.0001	<0.0001						
**1-MX**	*r*	0.08	0.07	0.94	0.91	0.91	0.83	1.00				
	*p*	0.0118	0.0206	<0.0001	<0.0001	<0.0001	<0.0001					
**1,3-DMX**	*r*	0.11	0.08	0.77	0.91	0.91	0.88	0.81	1.00			
	*p*	0.0006	0.0149	<0.0001	<0.0001	<0.0001	<0.0001	<0.0001				
**1,7-DMX**	*r*	0.12	0.10	0.78	0.86	0.91	0.88	0.86	0.93	1.00		
	*p*	<0.0001	0.0011	<0.0001	<0.0001	<0.0001	<0.0001	<0.0001	<0.0001			
**1,3,7-TMX**	*r*	0.14	0.08	0.69	0.81	0.89	0.92	0.75	0.90	0.90	1.00	
	*p*	<0.0001	0.0081	<0.0001	<0.0001	<0.0001	<0.0001	<0.0001	<0.0001	<0.0001		
**AAMU**	*r*	0.10	0.08	0.88	0.91	0.91	0.81	0.88	0.79	0.83	0.72	1.00
	*p*	0.0012	0.0094	<0.0001	<0.0001	<0.0001	<0.0001	<0.0001	<0.0001	<0.0001	<0.0001	

**Table 4:** Linear regression analysis of log-transformed urinary caffeine and caffeine metabolites and HOMA-IR and HOMA-β.

Urinary Metabolite Levels		HOMA-IR				HOMA-β ^1^			
		Unadjusted Model		Adjusted Model ^1^		Unadjusted Model		Adjusted Model ^1^	
		Beta ± SE	*p*	Beta ± SE	*p*	Beta ± SE	*p*	Beta ± SE	*p*
1-methyluric acid									
Per two-fold increase		0.13 ± 0.07	0.047	0.08 ± 0.08	0.293	3.88 ± 1.82	0.040	2.21 ± 1.97	0.269
Quartiles	Q1	Reference		Reference		Reference		Reference	
	Q2	−0.28 ± 0.30	0.348	0.00 ± 0.31	0.993	−17.08 ± 9.62	0.085	−4.65 ± 9.91	0.642
	Q3	0.03 ± 0.29	0.926	−0.08 ± 0.28	0.775	−2.83 ± 9.57	0.769	−1.79 ± 8.76	0.839
	Q4	0.72 ± 0.36	0.053	0.59 ± 0.41	0.164	18.99 ± 10.52	0.080	18.46 ± 12.04	0.135
1,3-dimethyluric acid									
Per two-fold increase		0.18 ± 0.07	0.008	0.16 ± 0.07	0.027	4.99 ± 1.62	0.004	4.23 ± 1.49	0.008
Quartiles	Q1	Reference		Reference		Reference		Reference	
	Q2	0.07 ± 0.24	0.790	0.40 ± 0.32	0.227	2.65 ± 8.00	0.743	13.12 ± 8.76	0.144
	Q3	0.67 ± 0.24	0.008	0.58 ± 0.32	0.084	16.05 ± 6.69	0.022	13.29 ± 7.75	0.096
	Q4	1.34 ± 0.37	0.001	1.39 ± 0.41	0.002	36.38 ± 10.11	0.001	34.02 ± 8.55	<0.001
1,7-dimethyluric acid									
Per two-fold increase		0.20 ± 0.05	0.001	0.18 ± 0.06	0.005	5.22 ± 1.52	0.002	4.08 ± 1.35	0.005
Quartiles	Q1	Reference		Reference		Reference		Reference	
	Q2	0.06 ± 0.29	0.825	0.35 ± 0.33	0.285	3.90 ± 6.80	0.570	12.37 ± 7.03	0.088
	Q3	0.51 ± 0.26	0.058	0.34 ± 0.29	0.249	8.88 ± 6.48	0.180	6.49 ± 8.03	0.425
	Q4	1.50 ± 0.37	<0.001	1.39 ± 0.42	0.002	40.66 ± 10.90	0.001	32.21 ± 8.95	0.001
1,3,7-trimethyluric acid									
Per two-fold increase		0.28 ± 0.07	<0.001	0.25 ± 0.07	0.001	7.24 ± 1.90	0.001	5.62 ± 1.59	0.001
Quartiles	Q1	Reference		Reference		Reference		Reference	
	Q2	0.39 ± 0.26	0.151	0.44 ± 0.28	0.125	19.35 ± 7.87	0.019	24.38 ± 7.64	0.003
	Q3	0.90 ± 0.32	0.008	0.72 ± 0.33	0.039	17.19 ± 8.44	0.050	17.11 ± 8.94	0.064
	Q4	1.83 ± 0.43	<0.001	1.55 ± 0.42	0.001	50.61 ± 12.84	<0.001	36.84 ± 9.86	0.001
1-methylxanthine									
Per two-fold increase		0.13 ± 0.05	0.012	0.10 ± 0.06	0.105	4.21 ± 1.53	0.010	2.42 ± 1.52	0.122
Quartiles	Q1	Reference		Reference		Reference		Reference	
	Q2	0.05 ± 0.32	0.879	0.41 ± 0.31	0.190	1.04 ± 8.73	0.906	14.86 ± 7.03	0.042
	Q3	0.41 ± 0.30	0.181	0.33 ± 0.29	0.255	8.12 ± 10.07	0.426	6.32 ± 7.65	0.415
	Q4	0.71 ± 0.33	0.037	0.60 ± 0.41	0.149	20.70 ± 9.58	0.038	14.08 ± 10.04	0.170
1,3-dimethylxanthine(theophylline)									
Per two-fold increase		0.23 ± 0.07	0.004	0.21 ± 0.07	0.007	5.85 ± 1.99	0.006	5.40 ± 1.50	0.001
Quartiles	Q1	Reference		Reference		Reference		Reference	
	Q2	0.24 ± 0.28	0.402	0.41 ± 0.26	0.119	12.44 ± 10.54	0.247	23.22 ± 9.35	0.018
	Q3	0.69 ± 0.27	0.017	0.83 ± 0.28	0.006	19.04 ± 9.09	0.044	26.44 ± 8.54	0.004
	Q4	1.47 ± 0.37	<0.001	1.26 ± 0.38	0.002	41.31 ± 11.94	0.002	34.15 ± 8.93	0.001
1,7-dimethylxanthine(paraxanthine)									
Per two-fold increase		0.25 ± 0.06	<0.001	0.21 ± 0.06	0.003	6.59 ± 1.79	0.001	5.73 ± 1.53	0.001
Quartiles	Q1	Reference		Reference		Reference		Reference	
	Q2	0.31 ± 0.28	0.276	0.45 ± 0.23	0.066	14.52 ± 9.06	0.119	22.21 ± 7.91	0.008
	Q3	0.81 ± 0.32	0.017	0.78 ± 0.31	0.017	13.30 ± 10.25	0.204	18.40 ± 8.78	0.044
	Q4	1.48 ± 0.34	<0.001	1.18 ± 0.37	0.003	43.64 ± 11.08	<0.001	34.56 ± 8.92	0.001
1,3,7-trimethylxanthine(caffeine)									
Per two-fold increase		0.26 ± 0.06	<0.001	0.24 ± 0.06	<0.001	5.53 ± 1.74	0.003	5.46 ± 1.57	0.002
Quartiles	Q1	Reference		Reference		Reference		Reference	
	Q2	0.27 ± 0.19	0.174	0.40 ± 0.16	0.018	8.57 ± 7.20	0.242	17.20 ± 7.52	0.029
	Q3	1.12 ± 0.33	0.002	1.11 ± 0.33	0.002	25.37 ± 9.19	0.009	28.51 ± 9.46	0.005
	Q4	1.51 ± 0.33	<0.0001	1.30 ± 0.32	<0.001	34.25 ± 10.58	0.003	31.59 ± 9.37	0.002
5-acetylamino-6-amino-3-methyluracil									
Per two-fold increase		0.20 ± 0.06	0.004	0.15 ± 0.07	0.037	5.37 ± 1.69	0.003	3.97 ± 1.62	0.020
Quartiles	Q1	Reference		Reference		Reference		Reference	
	Q2	0.13 ± 0.24	0.579	0.04 ± 0.27	0.874	4.11 ± 7.34	0.580	6.49 ± 7.75	0.408
	Q3	0.34 ± 0.24	0.164	0.13 ± 0.29	0.650	7.07 ± 6.21	0.263	7.01 ± 6.37	0.279
	Q4	1.21 ± 0.36	0.002	1.03 ± 0.38	0.011	34.36 ± 10.25	0.002	26.75 ± 9.32	0.007

^1^ Adjusted for age, sex, race, income, education, marital status, smoking history, alcohol consumption, physical activity, BMI, urinary creatinine clearance, and caffeine intake.

**Table 5:** Linear regression analysis of binary log-transformed urinary caffeine and caffeine metabolites and HOMA-IR and HOMA-β stratified by prediabetes status.

Urinary Metabolite Levels	Total	Normal (Fasting Plasma Glucose < 5.6 mmol/L)					Prediabetes (Fasting Plasma Glucose ≥ 5.6 mmol/L)				
		n	Unadjusted Model		Adjusted Model ^1^		n	Unadjusted Model		Adjusted Model ^1^	
			Beta ± SE	*p*	Beta ± SE	*p*		Beta ± SE	*p*	Beta ± SE	*p*
**HOMA-IR**											
1-MU	1036	577	0.07 ± 0.06	0.212	0.07 ± 0.05	0.118	459	0.12 ± 0.15	0.408	0.09 ± 0.18	0.645
1,3-DMU	1016	563	0.08 ± 0.05	0.125	0.09 ± 0.04	0.018	453	0.26 ± 0.13	0.053	0.24 ± 0.16	0.155
1,7-DMU	1020	565	0.11 ± 0.04	0.015	0.11 ± 0.03	0.001	455	0.24 ± 0.11	0.036	0.21 ± 0.12	0.099
1,3,7-TMU	980	542	0.14 ± 0.05	0.005	0.14 ± 0.04	0.001	438	0.39 ± 0.14	0.011	0.31 ± 0.14	0.039
1-MX	1036	577	0.09 ± 0.04	0.057	0.10 ± 0.03	0.004	459	0.14 ± 0.12	0.248	0.08 ± 0.13	0.549
1,3-DMX	1002	557	0.09 ± 0.06	0.156	0.10 ± 0.04	0.008	445	0.38 ± 0.16	0.021	0.33 ± 0.16	0.050
1,7-DMX	1019	565	0.14 ± 0.05	0.006	0.14 ± 0.03	0.000	454	0.30 ± 0.13	0.022	0.22 ± 0.13	0.101
1,3,7-TMX	994	548	0.13 ± 0.05	0.009	0.14 ± 0.04	0.001	446	0.30 ± 0.13	0.034	0.29 ± 0.12	0.028
AAMU	1027	571	0.10 ± 0.04	0.038	0.09 ± 0.04	0.016	456	0.27 ± 0.14	0.061	0.22 ± 0.18	0.236
**HOMA-B**											
1-MU	1036	577	4.69 ± 2.97	0.124	5.13 ± 3.08	0.105	459	3.47 ± 2.92	0.243	-0.61 ± 3.44	0.860
1,3-DMU	1016	563	5.02 ± 2.39	0.043	5.98 ± 1.72	0.002	453	5.64 ± 2.87	0.058	2.39 ± 3.09	0.445
1,7-DMU	1020	565	6.15 ± 2.16	0.008	6.27 ± 1.72	0.001	455	4.61 ± 2.54	0.079	1.72 ± 2.33	0.466
1,3,7-TMU	980	542	7.82 ± 2.27	0.002	7.59 ± 1.80	0.000	438	7.76 ± 3.44	0.031	4.20 ± 2.96	0.165
1-MX	1036	577	5.37 ± 2.34	0.028	5.99 ± 2.19	0.010	459	2.90 ± 2.34	0.224	-1.34 ± 2.38	0.578
1,3-DMX	1002	557	5.65 ± 2.66	0.041	6.79 ± 1.63	0.000	445	6.93 ± 3.52	0.057	4.11 ± 3.02	0.182
1,7-DMX	1019	565	7.58 ± 2.30	0.002	8.10 ± 1.76	<0.0001	454	5.96 ± 2.88	0.046	2.83 ± 2.46	0.258
1,3,7-TMX	994	548	7.10 ± 2.22	0.003	7.75 ± 1.86	0.000	446	4.43 ± 3.20	0.176	3.35 ± 2.69	0.222
AAMU	1027	571	5.21 ± 2.33	0.032	5.17 ± 2.07	0.018	456	6.39 ± 2.96	0.039	2.55 ± 3.31	0.446

^1^ Adjusted for age, sex, race, income, education, marital status, smoking history, alcohol consumption, physical activity, BMI, urinary creatinine clearance, and caffeine intake.
